# 532. Establishing a SARS-CoV-2 Monoclonal Antibody Infusion Clinic: Early Trends in Outcomes and Disparities

**DOI:** 10.1093/ofid/ofab466.731

**Published:** 2021-12-04

**Authors:** Allison J Hare, Judith M Rivera McPhaul, Patrick Cargan, Pablo Tebas, Kathleen Degnan, Jillian T Baron, Keith W Hamilton

**Affiliations:** 1 Perelman School of Medicine, University of Pennsylvania, North Oaks, Minnesota; 2 Clinical Practices of the University of Pennsylvania, Philadelphia, Pennsylvania; 3 Penn Medicine, Philadelphia, Pennsylvania; 4 Hospital of the University of Pennsylvania, Philadelphia, PA

## Abstract

**Background:**

SARS-CoV-2 monoclonal antibodies (SMA) have demonstrated efficacy in treatment of early, mild to moderate COVID-19 in patients at high risk for progression to severe COVID-19. We created an SMA infusion clinic at a large, urban academic medical center using both internal and community-based referral mechanisms to promote the equitable distribution of treatment.

**Methods:**

Data were analyzed from clinic referrals from December 13, 2020 through April 20, 2021. Patient demographics, census-based area deprivation index (ADI) scores (scale of 1-10, with 1 representing least socioeconomic deprivation and 10 representing most), and relevant comorbidities were collected. Outcomes included days of symptoms until referral, patient receipt of SMA therapy after referral, adverse events, and ER visits and hospitalizations within 14 days of SMA administration. Association between demographic factors and relevant outcomes were determined using chi-square or Wilcoxon rank-sum tests as appropriate.

**Results:**

47/433 (11%) referred patients were ineligible based on inclusion and exclusion criteria. Of eligible patients, 310/386 (80%) received treatment; patients who did not receive treatment either declined (93%), could not be contacted (5%), no-showed (1%), or were admitted for hypoxia (1%). Of treated patients, only 3 (1%) had adverse reactions. Within 14 days of SMA administration, 28 (9%) patients visited the ER or were admitted for COVID-19. Black patients had a longer median duration of symptoms prior to referral compared to White patients (5 vs. 3 days, *p* < 0.01) (Figure 1). White patients were more likely to receive SMA after referral compared to Black patients (88% vs. 64%, *p* < 0.01), as were patients with ADI score 1-5 compared to those with ADI score 6-10 (88% vs. 70%, *p* < 0.01) (Figures 2 and 3). Black patients who received SMA had a higher rate of ER visits or admissions than White patients, although the difference was not statistically significant (14% vs. 7%, *p* = 0.10).

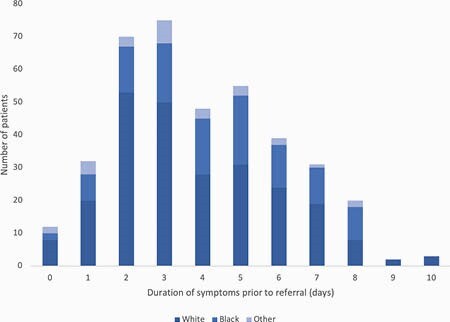

Figure 1. Bar graph displaying number of patients per race (White, Black, or Other) by duration of symptoms prior to referral.

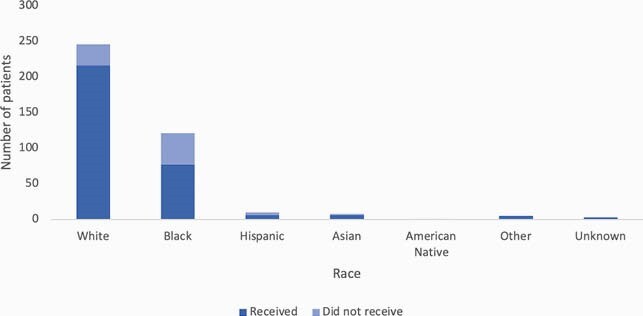

Figure 2. Bar graph displaying number of patients who did and did not receive SMA by race

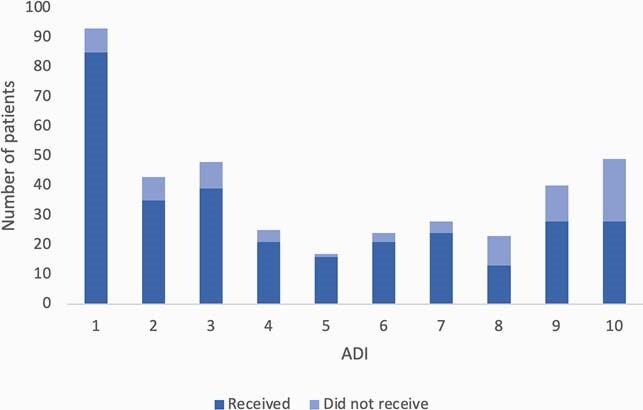

Figure 3. Bar graph displaying number of patients who did and did not receive SMA by ADI.

**Conclusion:**

Rate of adverse reactions and COVID-related ER visits or admissions were low in patients who received SMA. Despite efforts to promote the equitable distribution of treatment through multiple referral mechanisms, racial and socioeconomic disparities still exist.

**Disclosures:**

**All Authors**: No reported disclosures

